# DNA‐Inspired Multi‐Functional Double‐Cross‐Linking Self‐Healing Hydrogel Promotes the Healing of Diabetic Wounds

**DOI:** 10.1002/advs.202513784

**Published:** 2025-11-27

**Authors:** Pu Yang, Yue Hu, Yikun Ju, Naihsin Hsiung, Juyi Ye, Anquan Jian, Lanjie Lei, Bairong Fang

**Affiliations:** ^1^ Department of Plastic and Aesthetic (Burn) Surgery The Second Xiangya Hospital Central South University Changsha 410011 China; ^2^ Department of Plastic and Reconstructive Surgery Xijing Hospital Fourth Military Medical University Xi'an Shaanxi 710032 China; ^3^ Department of Dermatology The Second Xiangya Hospital Central South University Changsha Hunan 410011 China; ^4^ School of Mechanical and Electrical Engineering Central South University Changsha Hunan 410011 China; ^5^ Engineering & Design College Human Normal University Changsha Hunan 410011 China; ^6^ Key Laboratory of Artificial Organs and Computational Medicine of Zhejiang Province Shulan International Medical College Institute of Translational Medicine Zhejiang Shuren University Hangzhou Zhejiang 310015 China

**Keywords:** curcumin, diabetic wounds, exosomes, hydrogel

## Abstract

The impaired healing of diabetic wounds caused by microenvironmental imbalances presents a significant clinical challenge. In this study, a deoxyribonucleic acid (DNA)‐inspired, self‐healing hydrogel‐based delivery system—ATG@Exo^Cur^—is developed that synergistically promotes diabetic wound healing by modulating multiple pathological processes. This system features a double‐crosslinked, self‐healing hydrogel derived from natural polysaccharides, which leverages the principle of DNA base‐pairing to achieve controlled drug release and mechanical adaptability. Additionally, incorporating exosome‐loaded curcumin enhances its physicochemical stability and improves the targeted delivery efficiency. This study demonstrates that ATG@Exo^Cur^ effectively scavenges reactive oxygen species, suppresses chronic inflammation, promotes angiogenesis, and modulates the immune microenvironment by inducing macrophage polarization toward a pro‐repair phenotype. By integrating biomaterial engineering with biological activity, this system overcomes the limitations of monotherapies and offers a novel, highly efficient, and safe approach for treating chronic diabetic wounds.

## Graphical abstract

A self‐healing hydrogel crosslinked with dynamic nucleobase‐mimetic bonds delivers curcumin‐loaded exosomes. It promotes diabetic wound healing via a synergistic combination of antibacterial, anti‐inflammatory, pro‐angiogenic, and immunomodulatory mechanisms, providing a comprehensive therapeutic approach.

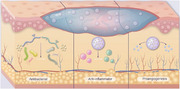

## 1 Introduction

Diabetic wounds, as one of the most common and severe complications of diabetes, not only significantly reduce the quality of life of patients but also impose a huge socio‐economic burden.^[^ ^1^, ^2^, ^3^, ^4^ ^]^ The pathological mechanism of diabetic wounds is highly complex, involving a multifactorial interplay of oxidative stress triggered by a persistently high‐glucose microenvironment, chronic inflammation, impaired angiogenesis, abnormal extracellular matrix remodeling, and immune dysregulation.^[^ ^5^, ^6^, ^7^, ^8^, ^9^ ^]^ While the current therapeutic approaches—such as dressing changes and the topical application of growth factors—can alleviate symptoms to a certain extent, they remain limited by prolonged healing times, a high risk of recurrent infections, and inadequate regulation of the wound microenvironment.^[^ ^10^, ^11^, ^12^ ^]^ Recently, novel hydrogels have achieved significant progress in promoting wound healing by targeting specific pathological pathways, such as modulation of macrophage phenotypes^[^ ^13^ ^]^ or scavenging excess reactive oxygen species (ROS).^[^ ^14^ ^]^ These findings highlight the immense potential of actively intervening in the wound microenvironment through material‐based approaches. However, due to the complexity of diabetic wounds involving multifactorial pathology, integrated therapeutic platforms capable of simultaneously coordinating multiple functions, including anti‐inflammatory, antioxidant, and pro‐angiogenic effects, are urgently required. In addition, there is an urgent need to develop advanced therapeutic strategies that can holistically address the pathological mechanisms underlying diabetic wounds and enhance the efficiency of tissue repair.

Recently, synergistic delivery systems of natural active ingredients and biomaterials have attracted considerable attention in the field of tissue repair. Curcumin (Cur), a polyphenolic compound derived from turmeric, exhibits significant potential for wound repair owing to its multiple biological functions.^[^ ^15^, ^16^, ^17^ ^]^ Cur can reduce inflammation by scavenging ROS and inhibiting the NF‐κB signaling pathway; however, its low water solubility, rapid metabolism, and photostability severely limit its bioavailability and clinical efficacy.^[^ ^18^, ^19^ ^]^ Exosomes (Exos) are a class of extracellular vesicles with a diameter of 30–150 nm that possess extensive biological functions.^[^ ^20^, ^21^, ^22^, ^23^ ^]^ Human adipose stem cell‐derived exosomes (ADSC‐Exo) not only have immunomodulatory functions similar to those of their parental cells but can also perform a wide range of tissue repair functions.^[^ ^24^, ^25^, ^26^ ^]^ More importantly, Exo can be used as natural nanocarriers of hydrophobic drugs (e.g., Cur) to enhance their therapeutic effect by improving drug solubility through their lipid bilayer structure.^[^ ^27^, ^28^, ^29^ ^]^

However, in vivo, Exos are rapidly cleared by the monocyte‐macrophage system, and free Exo^Cur^ has a short retention time in wounds, making it difficult to achieve a long‐lasting effect.^[^ ^30^, ^31^ ^]^ Self‐healing hydrogels are ideal drug delivery platforms for overcoming these limitations because of their injectability, adhesion, and dynamic cross‐linking properties.^[^ ^32^, ^33^ ^]^ However, most of the self‐healing covalent bonds (e.g., Schiff base bonds) exhibit insufficient performance, which makes it difficult to meet the long‐term application requirements of hydrogel dressings in diabetic wounds. The degradation of the hydrogel aligns well with the wound repair process, and its degradation products are non‐cytotoxic and biocompatible.

Here, inspired by the pairing of hydrogen bonds in DNA bases, we aimed to construct a double‐cross‐linked self‐healing hydrogel (ATG) by introducing DNA base complementary pairing bonds to traditional natural hydrogel biomaterial to enhance the performance deficiency of traditional natural self‐healing hydrogels. This hydrogel was constructed from adenine‐functionalized carboxymethyl chitosan (A‐CS) and thymine‐functionalized aldehyde‐functionalized hyaluronic acid (T‐AHA) and synergistically cross‐linked through dynamic Schiff base bonding and complementary base hydrogen bonding, which has several advantages. Compared to guanine–cytosine (G–C) pairing (three hydrogen bonds), adenine–thymine (A–T) pairing (two hydrogen bonds) exhibits moderate binding strength. This characteristic may enable it to better balance properties such as hydrogel stability, reversibility, and self‐healing when constructing dynamic materials. Thus, the biomimetic DNA double helix structure demonstrates better mechanical strength and rheological properties for simple Schiff base self‐healing hydrogels. This renders the hydrogel ideal for the dynamic mechanical conditions of the wound environment. Furthermore, its porous network enables efficient loading and slow release of Exo^Cur^, thereby extending the duration of drug action. Its exceptional stability and sustained‐release properties stem from the synergistic interaction between Schiff bases and A─T hydrogen bonds. Schiff bases provide permanent, covalent crosslinking points, ensuring the structural integrity of the hydrogel in physiological environments and preventing premature disintegration. The dynamic A─T hydrogen bond network plays two critical roles; mechanically, these bonds act as “sacrificial bonds,” dissipating energy through reversible breaking and reformation. This endows the hydrogel with superior toughness and self‐healing capacity to withstand dynamic mechanical stresses at the wound site. During mass transport, they collaborate with Schiff bases to form a denser and more intricate 3D network, significantly increasing resistance to water erosion and drug diffusion. In addition, the antibacterial, anti‐inflammatory, and moisturizing properties of CS and HA synergistically improve the microenvironment of diabetic wounds, effectively inhibit bacterial biofilm formation, and promote epithelial regeneration.

**Figure 1:**
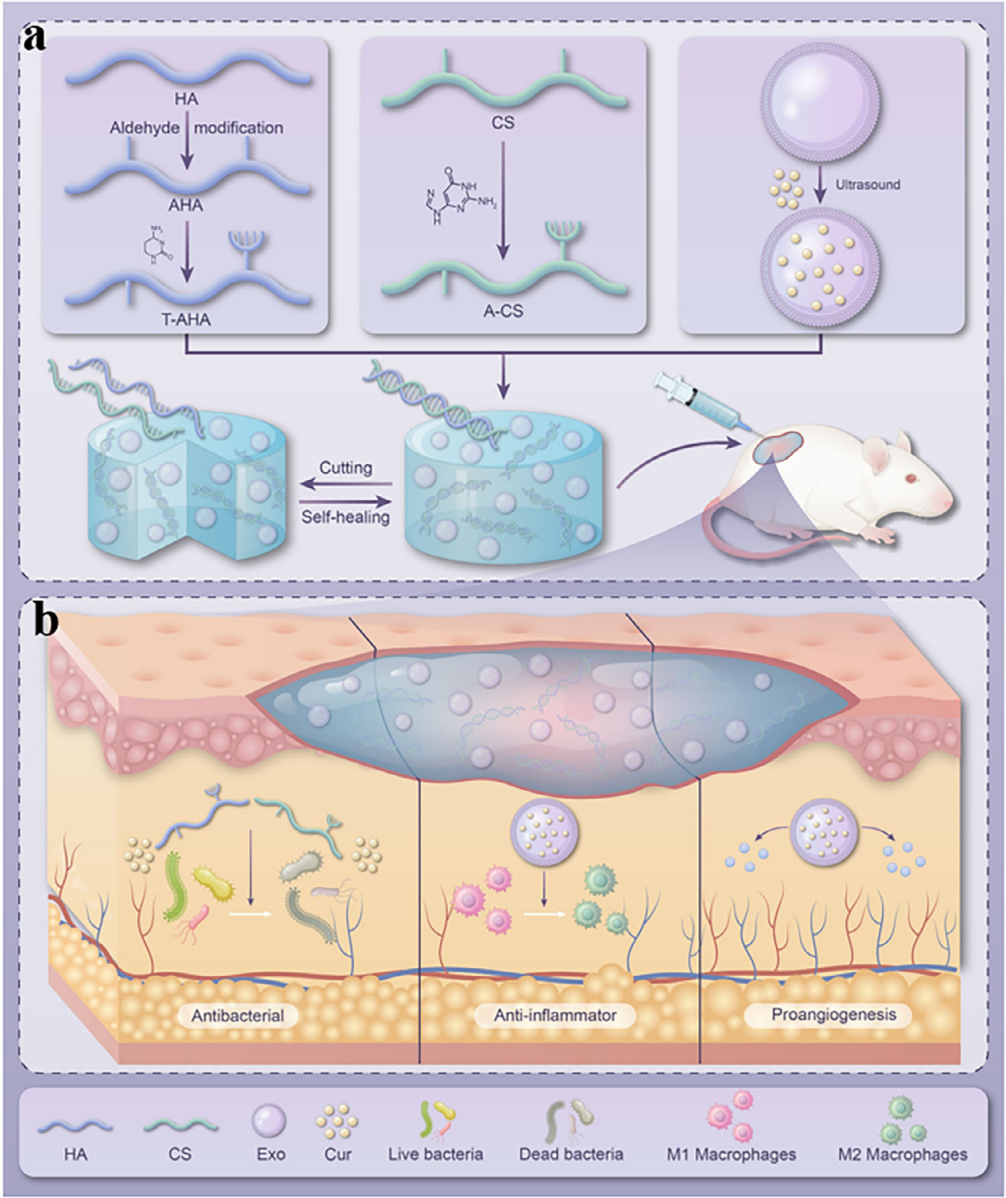
Schematic of the mechanism of DNA‐inspired multifunctional hydrogel promoting diabetic wound healing. a) Construction of double cross‐linked self‐healing hydrogels. b) Multifunctional hydrogel promotes the healing of diabetic wounds.

This study introduced a biomimetic strategy for the design of diabetic wound therapeutic materials (**Figure** **1**a), which not only provides an innovative solution for the efficient delivery of Exo^Cur^ but also overcomes the limitations of single therapeutic strategies by multimodal regulation of the wound microenvironment (antioxidant, anti‐inflammatory, pro‐angiogenic, and immune modulation) (Figure 1b). These findings are expected to provide a breakthrough strategy for the clinical treatment of chronic diabetic wounds, providing both a strong theoretical foundation and practical application value. They also provide a new paradigm for expanding the synergistic delivery system based on Exo^Cur^ in regenerative medicine.

## 2 Results and Discussion

### 2.1 Drug‐Carrying Properties and Characterization of Exo^Cur^

Cur was successfully loaded onto Exo by sonication. High‐performance liquid chromatography (HPLC) analysis revealed a loading rate of 23.1% and an encapsulation rate of 75.3%. These results are comparable to those obtained in previous studies that employed ultrasonication to prepare turmeric‐loaded engineered Exos. Moreover, Cur exhibited enhanced stability after Exo loading. On day seven, over 25% of Cur remained stable in Exo^Cur^ compared to that in unencapsulated Cur. Transmission electron microscopy (TEM) showed that the particle size of Exo^Cur^ was slightly larger than that of Exo. Nanoparticle tracking analysis (NTA) also showed that the particle size distribution of Exo^Cur^ was larger. Western blotting verified that Exo^Cur^ retained the expression of CD63, CD81, and TSG101, suggesting that the loading process did not disrupt the integrity of the Exo membrane (Figures S1 and S2, Supporting Information).

### 2.2 Physicochemical Properties of the Hydrogels

**Figure 2:**
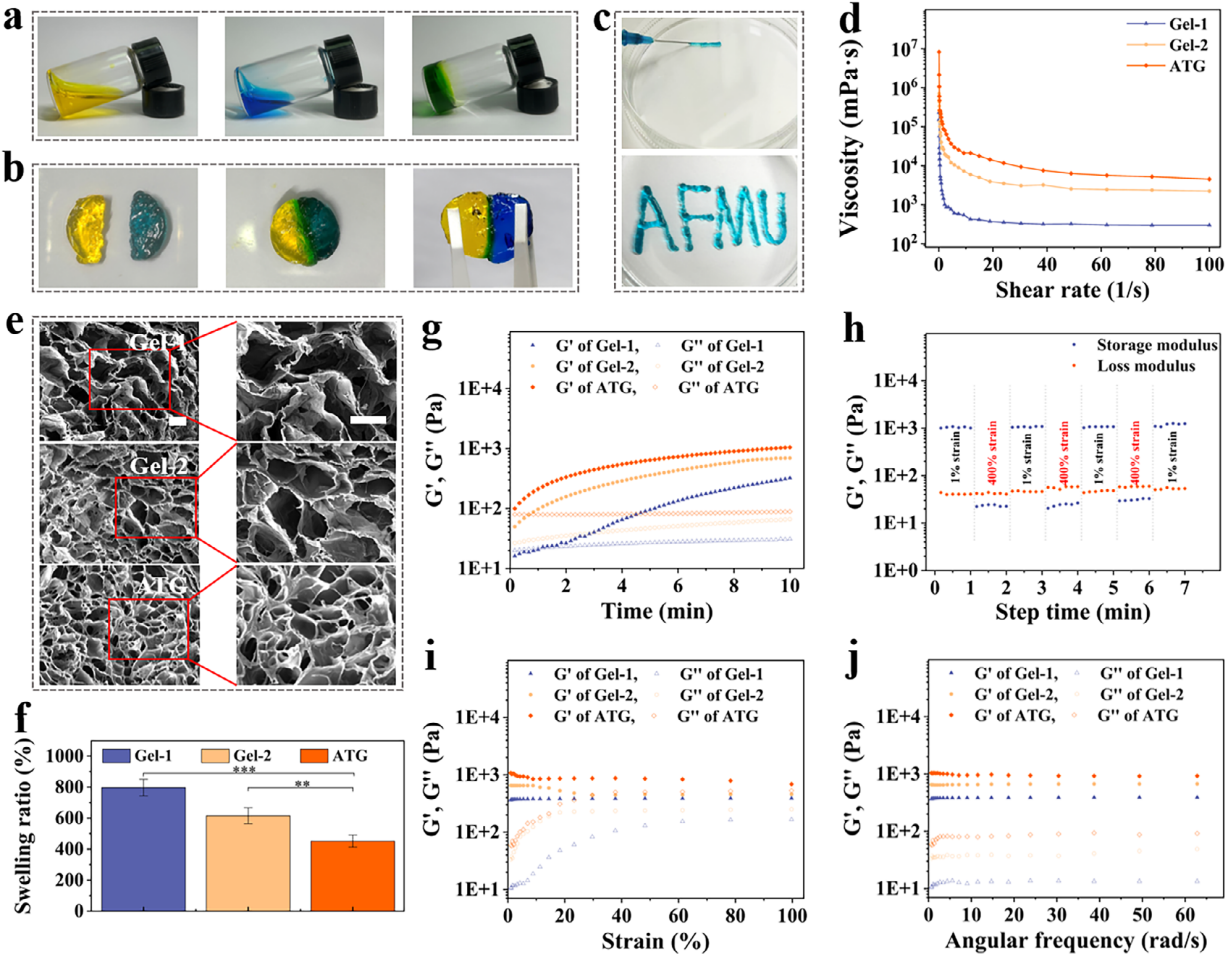
Characterization of ATG. a) Macroscopic synthesis of ATG hydrogel. b) Self‐healing properties of ATG. c) Injectability of ATG. d) Shear‐thinning properties of hydrogels. e) SEM results showing the microstructure of hydrogels under different crosslinking strengths. Scale bar *=* 100 µm. f) Assessment of swelling properties. g) Rheological properties under time scanning. h) Rheological properties under alternating strain. i) Rheological properties under amplitude scanning. j) Rheological properties under frequency scanning. (Hydrogel formulations: Gel‐1: 4% CS + 4% AHA, Schiff base crosslinking; Gel‐2: 5% CS + 5% AHA, Schiff base crosslinking; ATG: 5% A‐CS + 5% T‐AHA, Schiff base + A─T hydrogen bonding) (*n* = 3, Statistical differences: ^*^ *p* < 0.05, ^**^ *p* < 0.01, ^***^ *p* < 0.001, and ns, not significant).

Fourier transform infrared (FTIR) spectroscopy was used to detect HA and CS before and after continuous modification, respectively. Following aldehyde modification of HA, a new characteristic absorption peak appeared at 1731 cm^−1^ within its spectral range. This peak is attributed to the stretching vibration of the newly formed aldehyde group (─CHO), confirming the successful preparation of AHA. Subsequently, the product reacted with 1‐(carboxymethyl)thymidine (T─COOH). In the resulting T‐AHA spectrum, a distinct, broad characteristic peak appeared at 3275 cm^−1^, likely originating from the amide N─H stretching vibration of thymidine. Additionally, a new characteristic peak at 1340 cm^−1^ confirms the formation of a new covalent bond (C─N) during the graft reaction between AHA and 1‐(carboxymethyl)thymidine (T─COOH). This transformation clearly indicates that thymidine has been successfully grafted onto the AHA backbone (Figure S3, Supporting Information). Similarly, FTIR confirms the CS modification. Compared to pristine CS, A‐CS exhibits a new stretching vibration peak at 1117 cm^−1^ corresponding to the newly formed C─N bond, signifying successful CS modification. Concurrently, the characteristic peak near 1650 cm^−1^ in A‐CS becomes broader and more intense due to the introduction of (C═N/C═C) groups from adenine (Figure S4, Supporting Information).^[^ ^34^ ^]^ Compared to the gel, the N─H characteristic peak band of ATG exhibited a significant redshift from 3275 to 3255 cm^−1^, accompanied by broadening of the peak, confirming the formation of A─T hydrogen bonds in the ATG hydrogel. Concurrently, the characteristic peak at 1117 cm^−1^ (C─N) in the ATG sample further verifies the successful covalent incorporation of adenine into the hydrogel network (Figure S5, Supporting Information). Furthermore, titration analysis determined that the substitution degree of thymine in T‐AHA was 31.2%, while that of adenine in A‐CS was 25.3%. After the modified A‐CS and T‐AHA were dissolved, they were directly mixed to form a hydrogel (**Figure** **2**a). ATG Hydrogel shows good self‐healing and injectable ability (Figure 2b–d). To quantitatively demonstrate the critical role of hydrogen bonds in DNA base pairing for self‐healing performance, we employed urea to disrupt the hydrogen bond crosslinks within the ATG hydrogel. Following hydrogen bond disruption, the self‐healing time of the hydrogel increased from 28.1 ± 2.9 to 47.4 ± 5.3 min, confirming the essential contribution of hydrogen bonds to its self‐healing capability (Table S1, Supporting Information). Scanning electron microscope (SEM) images showed that the lyophilized hydrogel had a porous 3D structure (Figure 2e). The porosity of the hydrogel gradually decreases with increasing cross‐linking strength. The changes in porosity also affect the swelling rate of the hydrogel. With an increase in the cross‐linking strength, the porosity and swelling rate of the hydrogel decreased (Figure 2f; Figure S6, Supporting Information). Similarly, in hydrogel degradation and drug release experiments, double‐linked hydrogels exhibited slower degradation rates and more sustained drug release behavior (Figures S7 and S8, Supporting Information). Rheological tests showed that the hydrogels in all three cross‐linking conditions exhibited stable shear thinning capacity, which was consistent with the injectability of macroscopic characterization (Figure 2d). Rheological test results fully validated the superior properties of the dual‐crosslinked network. Regarding gel formation kinetics (Figure 2g), ATG not only exhibited the fastest rate of rise to the plateau phase in its storage modulus (G'), but also achieved a significantly higher final modulus value compared to Gel‐1 and Gel‐2. This indicates that dual crosslinking simultaneously enhances both the gel formation rate and ultimate mechanical strength. Notably, ATG exhibited a higher storage modulus than Gel‐2 at equivalent concentrations, proving this advantage stems from the crosslinking mechanism rather than a mere concentration increase. In the alternating strain cycling test (Figure 2h), after undergoing continuous cycling from 400% to 1% strain, ATG demonstrated near‐perfect modulus recovery capability, with self‐healing efficiency consistently maintained above 95%. This demonstrates that dynamic A─T hydrogen bonds, acting as “sacrificial bonds,” substantially enhance the network's energy dissipation and self‐healing capabilities. Furthermore, amplitude scanning results revealed that ATG exhibits a significantly higher critical strain value than both control groups, indicating that the dual‐crosslinked network possesses superior structural stability and deformation resistance (Figure 2i,j). The zeta potential test results show that all hydrogel samples exhibit a weak negative charge (Figure S9, Supporting Information), indicating that they can maintain a certain stability in skin wounds.

### 2.3 Antibacterial Properties of Hydrogels

**Figure 3:**
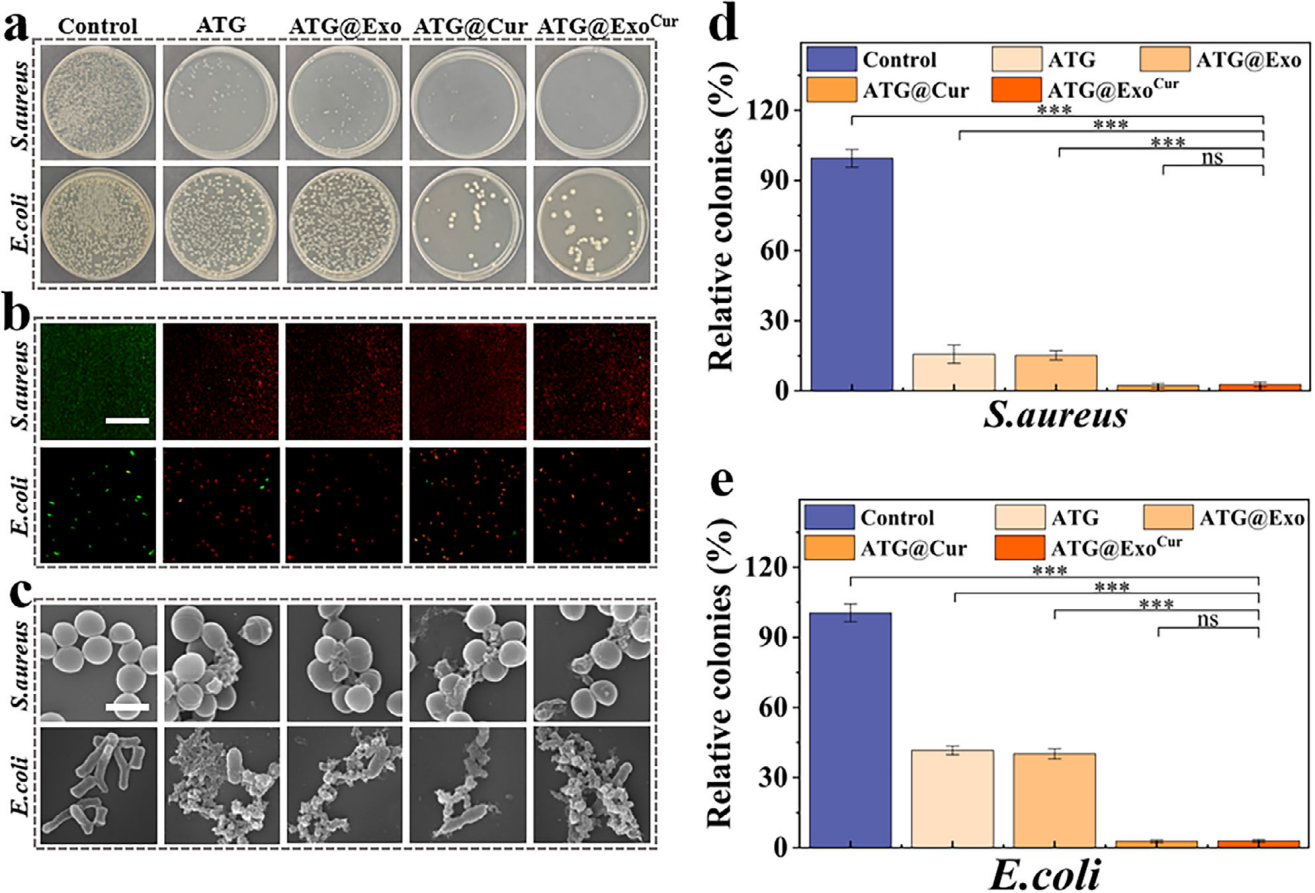
Evaluation of antimicrobial properties. a) Bacterial coating test results. b) Bacterial live death fluorescence staining results. Scale bar = 50 µm. c) Bacterial electron microscopy results. Scale bar = 1 µm. d) Quantitative statistics of the inhibitory effect on *S. aureus*. e) Quantitative statistics on the inhibitory effect on *E. coli*. (*n* = 3, Statistical differences: ^*^ *p* < 0.05, ^**^ *p* < 0.01, ^***^ *p* < 0.001, and ns, not significant).

Persistent bacterial infections in diabetic wounds (such as *Staphylococcus aureus* and *Escherichia coli* infections) may ultimately lead to chronic inflammation, hindering normal healing.^[^ ^35^, ^36^, ^37^ ^]^ The bacterial plate count results indicated that the ATG hydrogel itself effectively inhibits *Escherichia coli (E. coli) and Staphylococcus aureus (S. aureus)*. When Cur was loaded onto the hydrogel, its antibacterial efficacy was further enhanced, reducing the number of colonies by over 99% compared to the control group (**Figure** **3**a,d,e). The hydrogel exhibited stronger antibacterial activity against *S. aureus* (a Gram‐positive bacterium), primarily due to fundamental differences in the structure of the bacterial cell walls between the two types. The cell wall of Gram‐positive bacteria consists of a thick peptidoglycan layer. Despite its dense structure, it lacks an outer membrane for protection, making it more susceptible to positively charged antimicrobial components (such as cationic segments within the carboxymethyl chitosan backbone). These cationic polymers can be effectively adsorbed onto the negatively charged bacterial surface through electrostatic interactions, directly disrupting cell wall integrity and thereby exerting stronger antibacterial effects. Live/dead staining further confirmed the significant increase in the proportion of PI (red) positive bacteria (Figure 3b). SEM showed bacterial membrane rupture and wrinkling of wound morphology from hydrogel treatment, again indicating that ATG functioned synergistically with Cur to exert bactericidal effects through the cationic action of carboxymethyl chitosan (Figure 3c).

### 2.4 The Hydrogel Promotes Cell Migration and Angiogenesis

**Figure 4:**
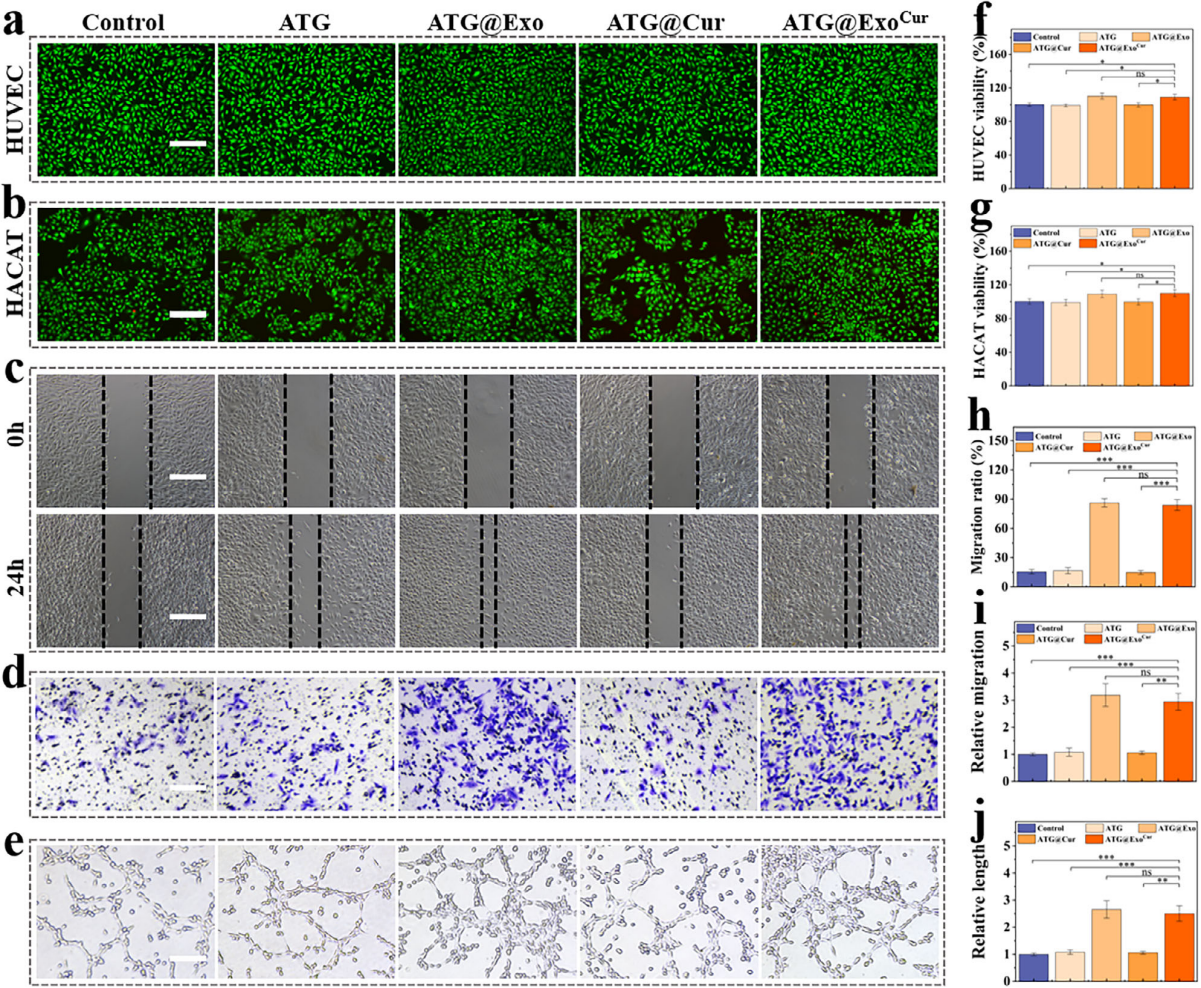
In vitro biological function validation. a) Results of HUVEC live‐death staining. b) HACAT cell live‐dead staining results. c) HUVEC cell scratch experiment. d) HUVEC transwell experiment. e) Angiogenesis experiments. f,g) Cytotoxicity of hydrogels. h,i) Migration‐promoting ability of hydrogels. j) Angiogenesis‐promoting ability of hydrogels. All scale bar = 200 µm. (*n* = 3, Statistical differences: ^*^ *p* < 0.05, ^**^ *p* < 0.01, ^***^ *p* < 0.001, and ns, not significant).

The high biocompatibility of the hydrogel is a core prerequisite for its clinical application. In vitro experiments showed that when treated with the hydrogel extract, human umbilical vein endothelial cells (HUVEC) and human keratin‐forming cells (HACAT) had a survival rate of more than 95%, and the hydrogel did not trigger hemolytic effects or organ toxicity. These results confirm its excellent biosafety (**Figure** **4**a,b,f,g; Figures S10 and S11, Supporting Information). Notably, when loaded with the Exo^Cur^ composite system, this hydrogel significantly promoted endothelial cell migration, increasing scratch closure rates by ≈60%, boosting Transwell migration cell counts by ≈2.5‐fold, effectively inducing longer vascularization lengths (Figure 4c,d,e,h–j). The dual effects of angiogenesis and migration on skin wounds effectively improved the endothelial dysfunction that commonly occurs in diabetic wounds, promoting neovascularization and nutrient supply.

### 2.5 Hydrogels Modulate Inflammation and Oxidative Stress

**Figure 5:**
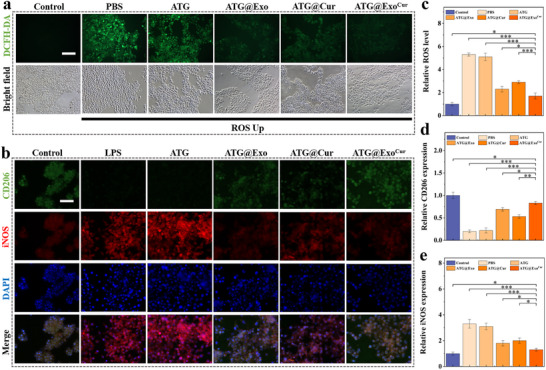
Anti‐inflammatory and macrophage‐modulating functions of hydrogels. a) ROS fluorescent probe experiment. b) Macrophage CD206/iNOS fluorescence double staining assay. c) Quantifying ROS levels. d) Hydrogels Modulate Macrophage CD206 expression. e) Hydrogels Modulate Macrophage iNOS expression. All scale bar = 100 µm. (*n* = 3, Statistical differences: ^*^ *p* < 0.05, ^**^ *p* < 0.01, ^***^ *p* < 0.001, and ns, not significant).

Chronic inflammation and oxidative stress are the core features of diabetic wounds and remain challenging during treatments. The phenolic hydroxyl structure of Cur in the hydrogel scavenged ROS (**Figure** **5**a,c). Additionally, treatment of HACAT with ATG@Exo^Cur^ extract reduced the intracellular ROS levels by 65% under oxidative stress conditions. The synergistic anti‐inflammatory and antioxidant effects disrupted the pernicious cycle of inflammation and oxidation in diabetic wounds and provided a stable microenvironment for tissue repair. This treatment significantly suppressed the lipopolysaccharide (LPS)‐induced M1 polarization of macrophages (with a roughly 50% reduction in iNOS‐positive cells) while promoting M2 polarization (with a nearly threefold increase in CD206‐positive cells) (Figure 5b,d,e). Additionally, STAT3 and STAT6 are key transcription factors regulating macrophage polarization. Immunofluorescence staining of phosphorylated proteins revealed that LPS stimulation promoted nuclear accumulation of p‐STAT3, while ATG hydrogel treatment induced significant nuclear translocation of both p‐STAT3 and p‐STAT6, consistent with previous findings.^[^ ^38^ ^]^ This co‐activation pattern aligns with the established model of M1‐to‐M2 phenotype conversion, which requires synergistic STAT3 and STAT6 signaling to suppress inflammatory signals and initiate reparative processes. These alterations in signaling pathways highly correlate with macrophage polarization markers (i.e., downregulation of iNOS and upregulation of CD206). Collectively, these findings indicate that ATG hydrogels drive macrophage polarization from M1 to M2 phenotypes through synergistic activation of STAT3 and STAT6 signaling pathways (Figure S12, Supporting Information). Compared to the latest exosome‐loaded hydrogel system reported by Wang et al., the composite hydrogel system developed in this study demonstrated superior promotion of cell migration and scratch healing.^[^ ^25^ ^]^ Similarly, the ROS scavenging capacity of our novel hydrogel system surpasses that of the previously reported gallic acid/exosome composite system, highlighting the synergistic antioxidant effects of Cur and Exos.^[^ ^39^ ^]^

### 2.6 ATG Hydrogel Promotes the Healing of Diabetic Wounds

**Figure 6:**
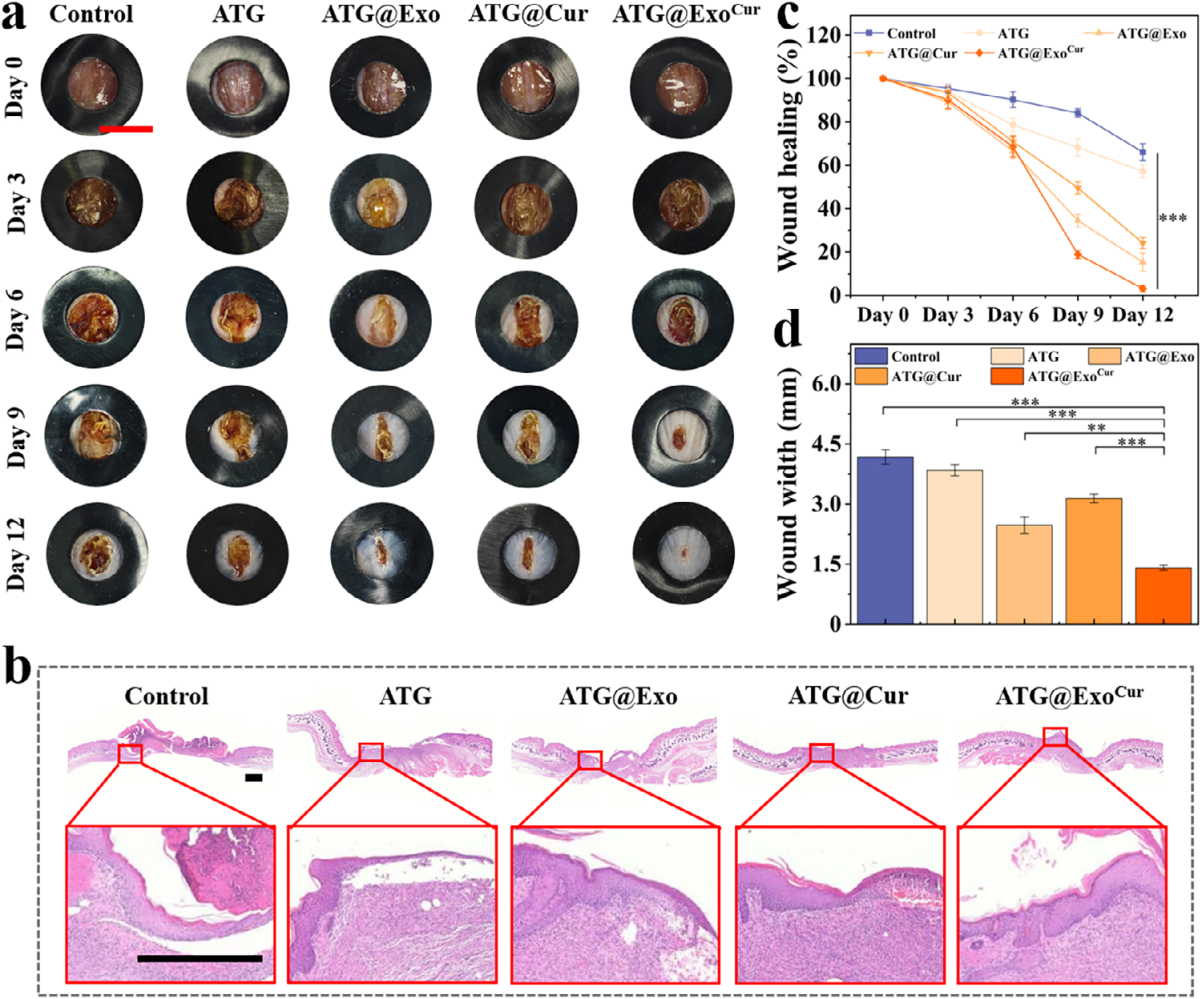
The hydrogel promotes diabetic wound healing. a) Continuous observation of wound healing in mice. Scale bar = 1 cm. b) H&E‐stained skin tissue of mouse wounds. Scale bar = 500 µm. c) Measurement of the wound healing rate in mice. d) Measure the wound width in mice. (*n* = 5, Statistical differences: ^*^ *p* < 0.05, ^**^ *p* < 0.01, ^***^ *p* < 0.001, and ns, not significant).

**Figure 7:**
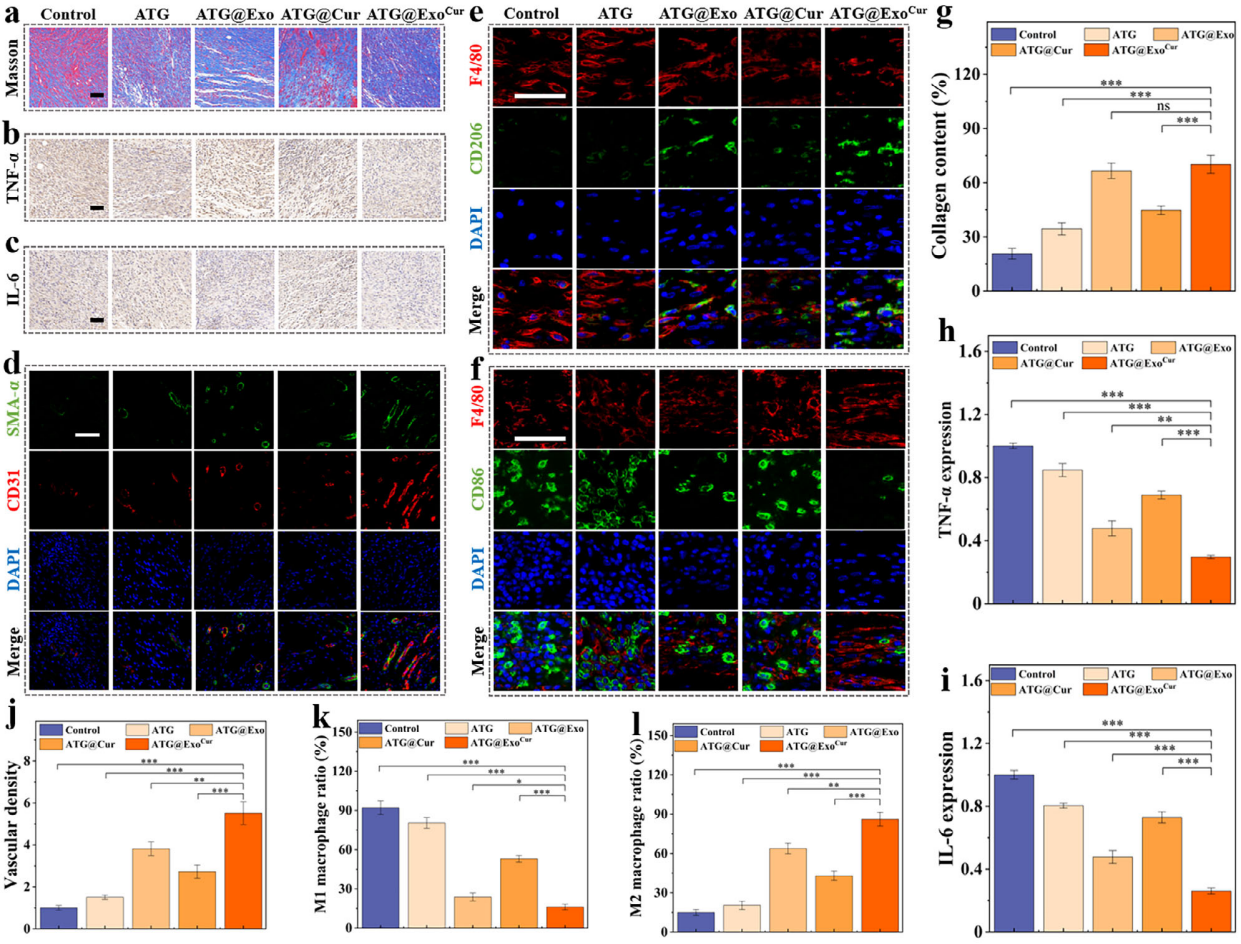
The hydrogel regulates inflammation and vascular regeneration of wound skin tissue. a) Representative Masson staining. b) Representative TNF‐α staining. c) Representative diagram of IL‐6 staining. d) Representative dual fluorescent staining of neovascularization. (α‐SMA, green; CD31, red). e) Representative M2 macrophage fluorescence double staining (F4/80, green; CD206, red). f) Representative high‐magnification images of M1 macrophage fluorescence double staining (F4/80, green; CD86, red). g) Differences in collagen deposition in skin tissue. h) Differences in TNF‐α expression. i) Differences in IL‐6 expression. j) Differences in relative vessel density. k) Differences in the proportion of M2 macrophages. l) Differences in the proportion of M1 macrophages. All scale bar = 100 µm. (*n* = 5, Statistical differences: ^*^ *p* < 0.05, ^**^ *p* < 0.01, ^***^ *p* < 0.001, and ns, not significant).

Animal studies demonstrated that wound healing in diabetic mice was significantly accelerated following hydrogel treatment. Compared to the PBS control group, the ATG@Exo^Cur^ treatment group exhibited a marked increase in the wound healing rate of ≈40% by day 12, accompanied by a corresponding significant reduction in wound width (**Figure** **6**a,c,d). H&E staining showed complete epidermal regeneration after hydrogel treatment with the composite group (ATG@Exo^Cur^), and Masson staining showed a dense arrangement of collagen fibers (Figures 6b, **7**a,g). We performed quantitative analysis of inflammatory cell infiltration in the dermis. The control group exhibited the highest cell density, indicating a significant inflammatory response. In contrast, the ATG@Exo^Cur^ group showed a marked reduction in cell density within the wound area (Figure S13, Supporting Information). And, the expression of TNF‐α and IL‐6 in the ATG@Exo^Cur^ group was significantly decreased (Figure 7b,c,h,i). Immunofluorescence double‐standard analysis further confirmed that the amount of neovascularization (CD31/α‐SMA) in this group was significantly increased (Figure 7d,j). The proportion of M1 macrophages was significantly reduced, and that of M2 macrophages was significantly increased (Figure 7e,f,k,l). The above experimental results are also similar to the relevant cell experiment results. However, it is worth noting that the simple ATG hydrogel and the control group also showed significant low expression of anti‐inflammatory factors, low distribution of M1 macrophages, and neovascular reconstruction, which may be related to the hydrogel's protection of the wound and its antibacterial effects. Therefore, the direct protective and antibacterial effects of the hydrogel on the wound cannot be ignored. More importantly, the potent immunomodulatory outcomes are intrinsically linked to the unique design and multimodal functionality of the ATG@Exo^Cur^ hydrogel. The dual‐crosslinked ATG network provides a sustained release profile, ensuring long‐term availability of Exo^Cur^ within the wound microenvironment. First, the active antimicrobial components within the hydrogel directly treat bacterial infections in diabetic wounds, preventing further exacerbation of bacterial‐induced wound inflammation by eliminating pathogens. Subsequently, the Cur component alleviates oxidative stress by scavenging ROS—a key driver of M1 polarization—thereby creating a favorable environment for phenotypic conversion. Furthermore, ADSC‐derived exosomes deliver specific immunomodulatory substances (e.g., miRNAs, cytokines), whose synergistic effects actively regulate macrophages, promoting their polarization toward the pro‐healing M2 phenotype.

These results again suggest that the hydrogel can regulate inflammatory responses by regulating macrophage polarization and accelerate wound healing by promoting angiogenesis. In the field of hydrogels for diabetic wound repair, hydrogel functionality is predominantly optimized for a single specific function. For instance, hydrogel systems loaded with MoS_2_/CaO_2_ nanoenzymes can effectively scavenge ROS but lack functions such as promoting angiogenesis and regulating macrophages. Conversely, the immunomodulatory hydrogel developed by He et al., while capable of efficiently clearing ROS and modulating macrophage polarization, lacks antimicrobial properties. In contrast, the hydrogel developed in this study integrates four functions: antibacterial, antioxidant, pro‐angiogenic, and immunomodulatory. It achieved a 98% healing rate within 12 days, outperforming the aforementioned single‐function systems.

These findings reaffirm that hydrogels can synergistically accelerate diabetic wound healing by simultaneously regulating macrophage polarization and angiogenesis. However, existing studies predominantly focus on single functions: MoS_2_/CaO_2_ nanozyme hydrogels excel at ROS scavenging but lack pro‐angiogenic and immunomodulatory capabilities.^[^ ^40^ ^]^ While the immunomodulatory hydrogel by He et al. can scavenge ROS and induce M2 polarization, it lacks antibacterial activity.^[^ ^41^ ^]^ In contrast, the hydrogel developed in this study integrates four functions—antibacterial, antioxidant, pro‐angiogenic, and immunomodulatory—achieving a 98% healing rate in just 12 days, significantly outperforming the aforementioned single‐function systems. Further comparative analysis reveals: Exosome‐loaded hydrogels typically require 14 days or longer to achieve >90% wound healing rates in diabetic mice, with lower absolute healing areas than this study.^[^ ^42^, ^43^ ^]^ When using exosomes alone (whether plant‐derived^[^ ^44^ ^]^ or mesenchymal stem cell‐derived^[^ ^45^ ^]^) without suitable hydrogel carriers, healing periods extend to 16–21 days. Thus, this strategy achieved a breakthrough in both therapeutic efficacy and timeliness compared to similar approaches.

## 3 Conclusion

In this study, we developed a novel DNA‐inspired, self‐healing hydrogel, ATG@Exo^Cur^, designed to achieve multidimensional regulation of the diabetic wound microenvironment by integrating the biological functions of Exos with the engineering advantages of hydrogels. Experimental analyses demonstrated that ATG@Exo^Cur^ enables the sustained release of bioactive Exo^Cur^, significantly promoting vascular neogenesis, reducing oxidative stress, and inducing macrophage M2 polarization, thereby accelerating wound healing. This system not only offers an innovative strategy for the clinical treatment of chronic diabetic wounds but also establishes a theoretical foundation for the application of ATG@Exo^Cur^‐based synergistic delivery systems in regenerative medicine. Moreover, this study did not construct hydrogels based on long‐chain DNA, representing a fundamental departure from traditional DNA hydrogel design principles. We innovatively introduce individual adenine and thymine bases as side chains into a natural polysaccharide backbone, leveraging their specific molecular recognition capabilities to form a dynamic hydrogen‐bond network. This approach offers the advantages of low cost and high safety. Future research should prioritize clinical translation, including process optimization, large‐scale production, and multicenter clinical trials, to facilitate its integration into clinical practice.

## 4 Experimental Section

### Materials

Various chemical reagents came from McLean Biotechnology (Shanghai, China). All types of cell experiment‐related reagents and kits were sourced from ServiceBio (Wuhan, China). HUVEC, HACAT and Human adipose stem cells were purchased from Procell (Wuhan, China). 8‐week‐old female C57BL/6J mice were purchased from Slaughter Kingda Laboratory Animal Co. The Ethics Committee for Animal Experimentation of the Second Xiangya Hospital of Central South University approved all the animal experimental procedures (approval number: 20250771).

### Preparation and Characterization of Exo^Cur^

Supernatants from the 3rd to 6th passages of well‐growing ADSCs were collected and subjected to exosome extraction via differential ultracentrifugation. The specific procedure involved sequential centrifugation at 300 × g, 2000 × g, and 10 000 × g to progressively remove cells and debris. Finally, a 70  min ultracentrifugation at 100 000 × g yielded a purified exosome pellet, which was resuspended in PBS for subsequent experiments.

Based on the methods described in previous literature, Cur (Sigma–Aldrich) was loaded onto the Exo using sonication.^[^ ^16^, ^46^ ^]^ Exos were mixed with Cur in a 1:2 mass ratio in PBS. The sample was ultrasonically treated using an ultrasonic homogenizer (Scientz‐IID) at 200 W power in pulse mode (2 s on, 2 s off) for a total duration of 3 min. After sonication, the sample was placed in an ice bath for 3 min, followed by incubation of the exosomes at room temperature for 30 min to allow complete recovery and stabilization of the exosomal membrane structure. Finally, the processed mixture underwent ultracentrifugation (100 000 × g, 70 min). The drug loading rate was determined using HPLC (Agilent 1260 Infinity II), encapsulation rate (%) = (mass of Cur in Exo^Cur^/mass of initial Cur) × 100%. Additionally, to further evaluate the stability of Exo^Cur^, both Exo^Cur^ and free Cur were placed in PBS (pH 7.4). The absorbance of the samples was measured periodically at 420 nm to assess sample stability. TEM (Hitachi HT7800), NTA (Malvern NanoSight NS300), and western blotting (for Calnexin, CD81, TSG101, and CD63) were used to characterize Exo^Cur^.

### Hydrogel Synthesis and Characterization

Adenine‐functionalized CS was prepared using a previously reported method. Specifically, after CS dissolution, 3‐(9‐adenyl)‐propionic acid (A─COOH) was added, and the carboxyl group was activated by EDC/NHS, followed by dialysis and purification after 48 h at room temperature to obtain A‐CS. Thymine‐functionalized aldehyde‐functionalized hyaluronic acid was prepared. First, hyaluronic acid (MW = 100 kDa) undergoes aldehyde modification and was subsequently coupled with 1‐(carboxymethyl)thymine (T─COOH) to obtain T‐AHA.^[^ ^34^, ^47^ ^]^ The resulting product was freeze‐dried. FTIR (Thermo Nicolet iS50) was performed to detect characteristic peaks to demonstrate the success of the reaction. The degree of A‐CS substitution was determined by measuring changes in the amino content of the polymer before and after modification. Specifically, precisely weighed samples of modified and unmodified polymers were dissolved separately in excess 0.1 m HCl. Subsequently, back titration with 0.1 m NaOH was performed, and the pH curve was recorded. The degree of substitution was calculated by determining the difference in the volume of NaOH consumed during neutralization. Similarly, the degree of substitution for T‐AHA was evaluated by measuring the changes in aldehyde content using the hydroxylamine method.

First, CS and AHA were dissolved at a ratio of 4:4 (w%/w%) and 5:5 (w%/w%), respectively, and the self‐healing hydrogels were named Gel‐1 and Gel‐2. Then, A‐CS and T‐AHA were cross‐linked at a rate of 5:5 (w%/w%) by dynamic Schiff base bonds (aminoaldehydes) and base‐complementary hydrogen bonds to form ATG. The zeta potential of the hydrogel was measured using Zetasizer Ultra (Malvern Panalytical). The rheometer (TA DHR‐2) was used to measure changes in hydrogel moduli under different scanning modes, including the storage modulus (G′) and loss modulus (G″). Next, the self‐healing efficiency was calculated. During the first strain cycle, the initial storage modulus (G′initial) was recorded, along with the stable storage modulus (G′final) at the end of the recovery phase following failure at 400% large strain. The self‐healing efficiency (%) was calculated via the following formula = (G′final / G′initial) × 100%.

Hydrogel samples with weights of W0 were extracted. The hydrogel sample was placed in PBS. At a specific time point, the hydrogel was removed, dried, and weighed as the sample (wt). The degradation ratio was calculated via the following formula: (W0‐Wt)/ W0 × 100%. The hydrogels were freeze‐dried, and the hydrogel samples were cut into specimens of specific shapes and sizes and accurately weighed to a constant dry weight (Wd) after absorbing the surface moisture with filter paper. Then, the sample was weighed repeatedly after absorbing PBS and measuring the wet weight (Ws). The swelling ratio was calculated using the following formula: (Ws‐Wd)/ Wd × 100%. The structure of freeze‐dried hydrogels using an SEM was then observed. The porosity of the hydrogels was calculated under different crosslinking conditions. The release of exosomes from hydrogels was determined using the BCA assay kit (Sigma–Aldrich).

Cylindrical hydrogels (10 mm diameter, 5 mm height) were completely severed and immediately rejoined, with the time required for complete fusion recorded. To validate DNA hydrogen bonding, the same test was performed on ATG hydrogels after soaking in 4 m urea solution for 4 h.

### Evaluation of the Antimicrobial Performance of the Hydrogels

*E. coli* and *S. aureus* were chosen to evaluate the antimicrobial properties of the hydrogel. First, cultivate the bacteria using Luria Bertani (LB) medium. Subsequently, the bacterial suspension with the hydrogel samples for 12 h was co‐cultured. At the end of co‐cultivation, the bacterial suspension was diluted 1 × 10^5^ times and inoculated 100 µL of the bacterial solution onto each LB solid medium. Invert the culture medium and incubate for 12 h, then count the bacterial colonies.

In addition, the co‐cultured bacteria were fixed, then dehydrated using a gradient concentration of ethanol solution, followed by spraying with tert‐butanol. Finally, an observation was conducted using SEM.

Live/dead staining experiments were performed on bacteria. Specifically, the co‐cultured bacterial suspension was stained for 20 min with propidium iodide (PI) and SYTO‐9, two dyes that label dead and live bacteria, respectively. After staining, the live/dead states of the bacteria were observed using orthogonal fluorescence microscopy.

### Biocompatibility Test of Hydrogels

After seeding the cells into 48‐well plates, the cytotoxicity of the hydrogel using the CCK‐8 kit and live/dead cell staining was evaluated. In addition, the biocompatibility of the ATG hydrogels was tested using a hemolysis assay. A 10% erythrocyte suspension was prepared by washing mouse erythrocytes with PBS. Subsequently, collect 500 µL red blood cell suspension and incubate it with the liquids from different experimental groups. After incubating, the hemolysis rate of the supernatant was calculated using a spectrophotometer. Finally, at the end of the animal experiments, different visceral tissues were subjected to H&E staining to assess organ toxicity.

### Detection of the Pro‐Proliferation Ability of Hydrogels

First, HUVEC were seeded into six‐well plates and cultured until cell fusion occurred. After the HUVEC were scratched, they were co‐cultured with the hydrogel leachate to observe cell migration at different time points. Second, by stimulating HUVEC in Transwell chambers using hydrogels from different experimental groups and counting the number of cells that migrated through the membrane after 24 h via staining, the hydrogels' promotion of cell migration was further confirmed. Additionally, HUVEC were seeded onto Matrigel, co‐cultured with hydrogel filtrate from different groups, and vascular network formation was observed.

### Detection of the Anti‐Inflammatory and Antioxidant Capacity of the Hydrogels

RAW264.7 cells were stimulated with 1 µg mL^−1^ LPS and treated with hydrogel leachate for 24 h. Following cell fixation, permeabilize the cells according to the kit instructions. Subsequently, cells were incubated with iNOS, CD206, p‐STAT3, and p‐STAT6 antibodies, respectively. Following secondary antibody staining, cellular protein expression was visualized via fluorescence microscopy.

In addition, HACAT cells were seeded into 12‐well plates to establish a ROS cell model. After subjecting the cells to different treatments, the intracellular ROS levels were measured using a fluorescent probe. The intracellular fluorescence intensity was observed using fluorescence microscopy.

### In Vivo Treatment with the Hydrogels

Based on the methods described in previous literature,^[^ ^48^ ^]^ C57BL/6J mice (weighing 20–25 g) were administered a fresh daily dose of 50 mg kg^−1^ streptozotocin (STZ) solution in 0.1 m sodium citrate buffer (pH *4.5*) via intraperitoneal injection for 14 consecutive days following a 6 h fast. Fasting blood glucose levels were measured using a blood glucose meter via tail vein blood sampling. Mice exhibiting stable blood glucose readings ≥16.7 mmol L^−1^ were deemed successful diabetic models and included in subsequent experiments. After confirming successful diabetic wound modeling, the mice were anesthetized. Hydrogels were used to treat full‐thickness skin wounds (1 cm diameter) on the backs of mice, with PBS‐containing hydrogel dressings applied to mice from different experimental groups. Wound healing was continuously documented using a digital camera over 12 days.

Paraffin‐embedded tissue sections were prepared from the wound site and examined using H&E staining and Masson's trichrome staining to assess skin tissue healing, inflammatory cell infiltration, and collagen deposition. Immunohistochemical staining (TNF‐α and IL‐6) was used to assess wound inflammation. Immunofluorescence double‐label staining (CD31/α‐SMA, F4/80 co‐staining with CD86 for M1 macrophages, F4/80 with CD206 for M2 macrophages) was used to quantitatively analyze the angiogenesis and macrophage polarization phenotype to evaluate the pro‐angiogenesis effect and regulatory effect of hydrogels on chronic inflammation.

### Statistical Analysis

Statistical analysis was performed using SPSS software (version 17.0). Data were expressed as the mean ± standard deviation (*n* ≥3). Comparisons among multiple groups were conducted by one‐way ANOVA followed by Dunnett's post hoc test. Statistical significance is expressed as follows: ^*^ *p* < 0.05, ^**^ *p* < 0.01, ^***^ *p* < 0.001, ns: not significant.

## Conflict of Interest

The authors declare no conflict of interest.

## Supporting information



Supporting Information

## Data Availability

The data that support the findings of this study are available from the corresponding author upon reasonable request.
