# Supplementary material for: DNA‐Inspired Multi‐Functional Double‐Cross‐Linking Self‐Healing Hydrogel Promotes the Healing of Diabetic Wounds
Source: Adv Sci (Weinh). 2025 Nov 27;13(8):e13784. doi: 10.1002/advs.202513784 (PMC12884814; doi:10.1002/advs.202513784)
Supplement: Supplementary file 1 — Supporting Information [file ADVS-13-e13784-s001.docx]

Supporting Information

**DNA-inspired multi-functional double-cross-linking self-healing hydrogel promotes the healing of diabetic wounds**

**Pu Yang, Yue Hu, Yikun Ju, Naihsin Hsiung, Juyi Ye, Anquan Jian, Lanjie Lei*, Bairong Fang ***


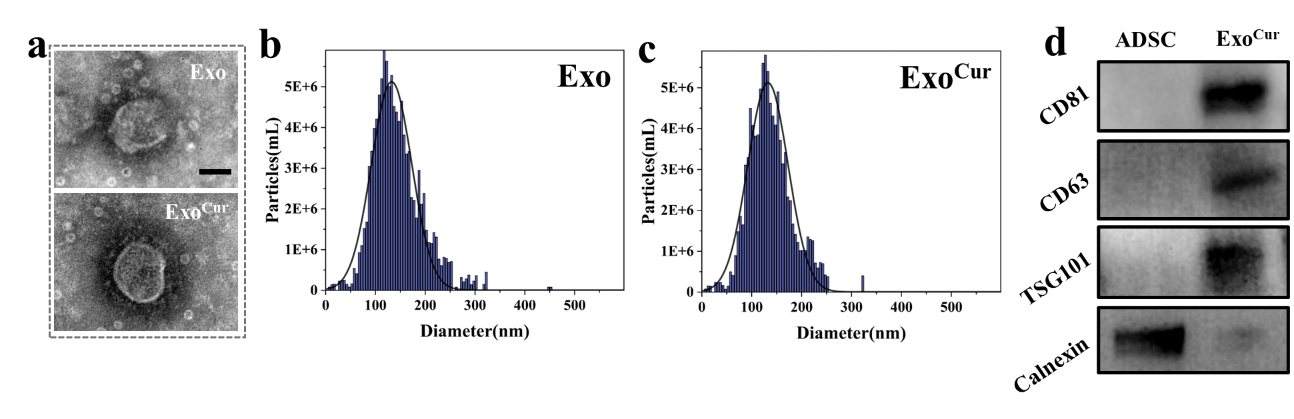


**Figure S1.** (a) Transmission electron microscopy to observe the morphology of exosomes. Scale bar is 100 nm. (b) Particle size analysis of Exo. (c) Particle size analysis of Exo^Cur^. (d) Western blotting of the Exo^Cur^ maker protein.


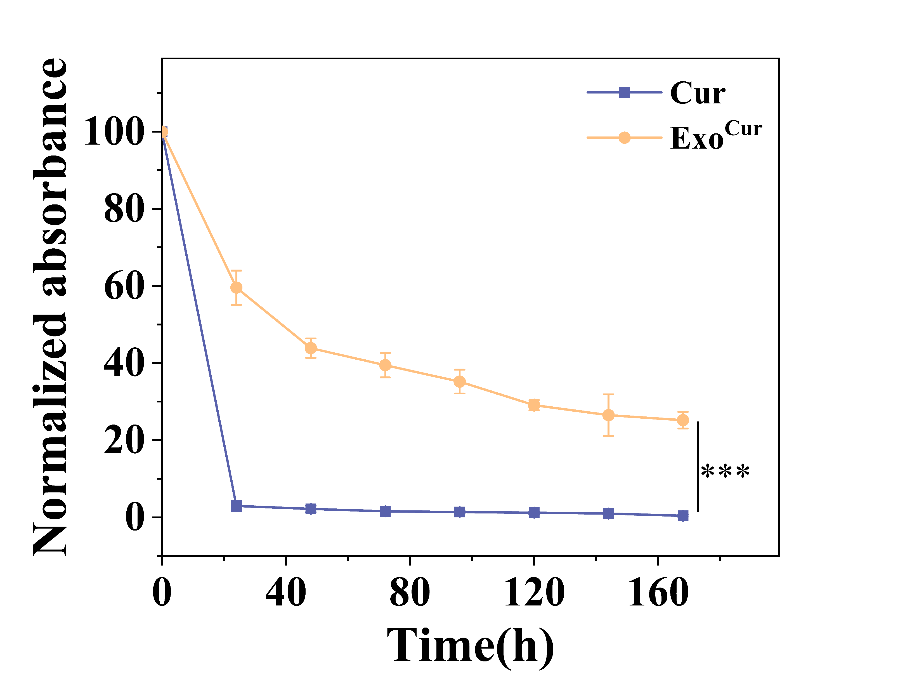


**Figure S2.** Stability of Exo^Cur^.


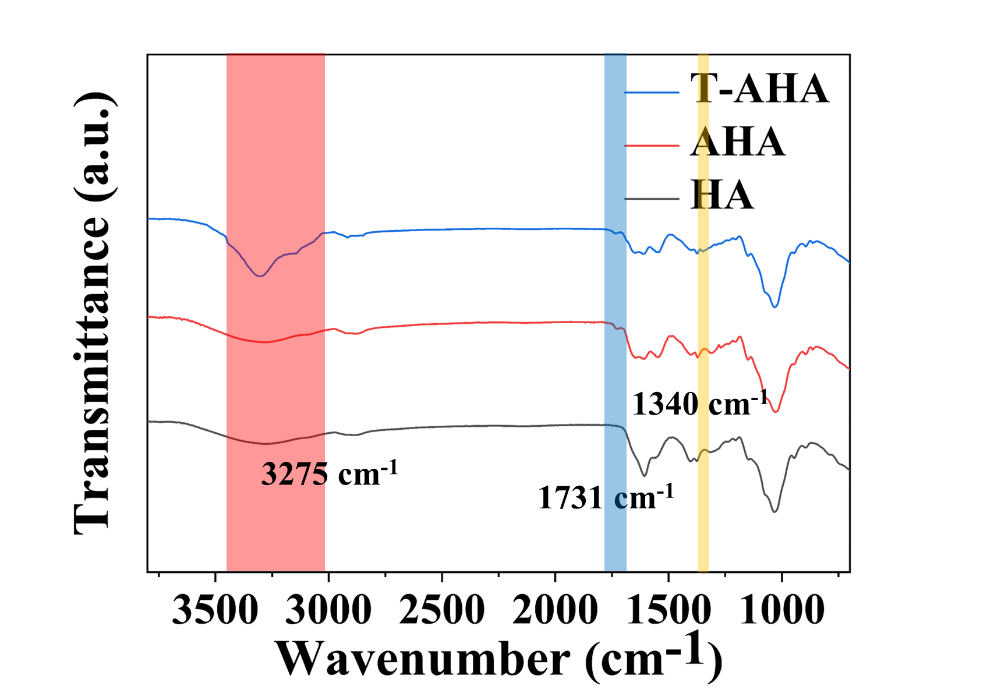


**Figure S3.** Fourier transform infrared spectra of HA, AHA and T-AHA.


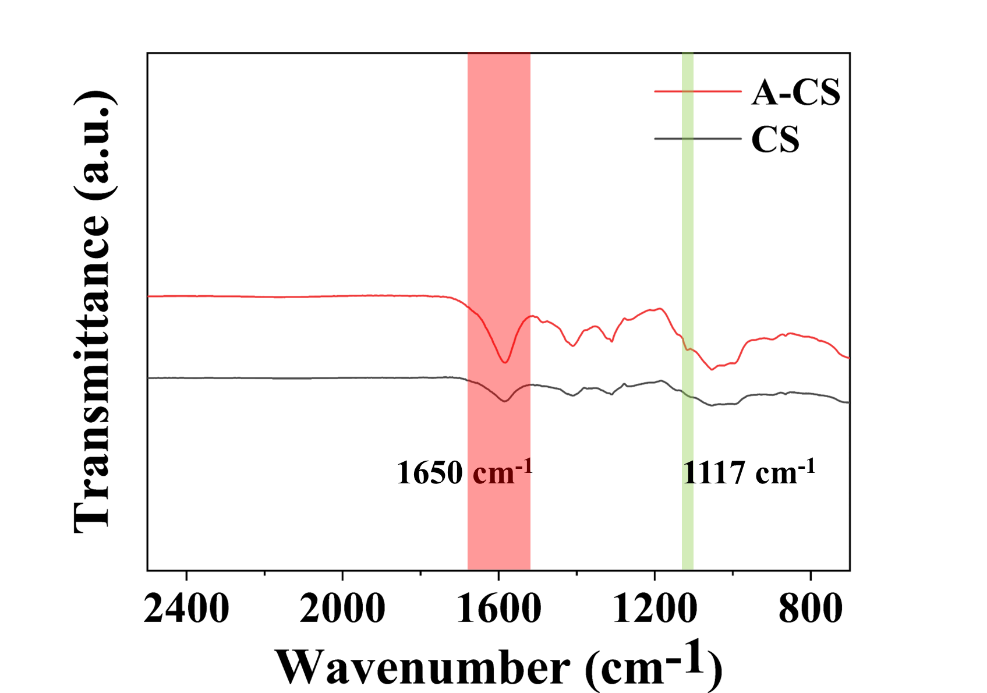


**Figure S4.** Fourier transform infrared spectra of CS and A-CS.

**
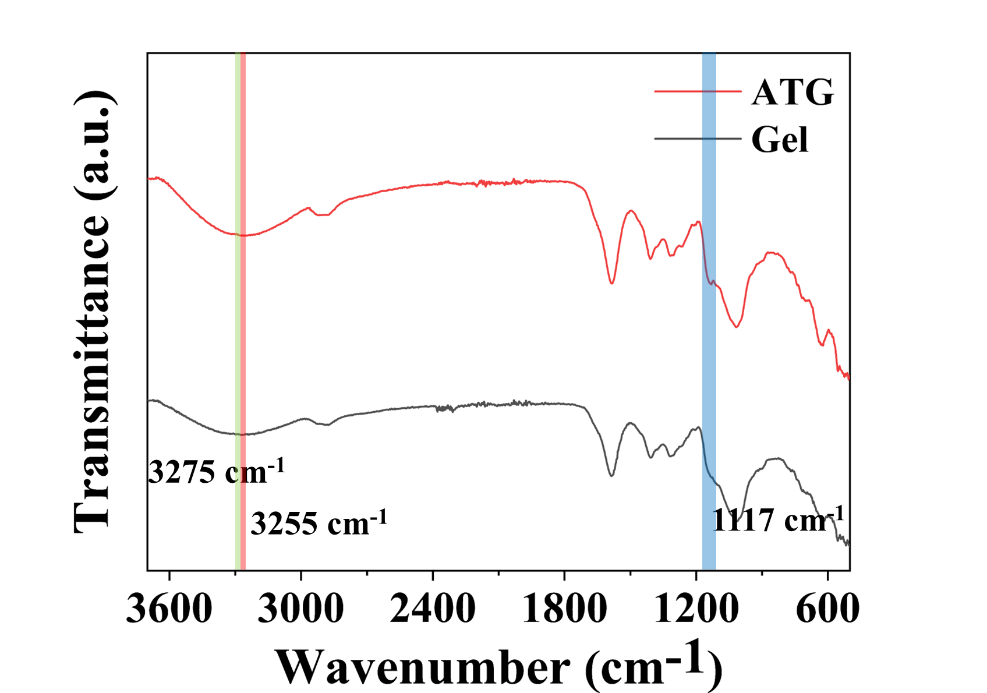
**

**Figure S5.** Fourier transform infrared spectra of Gel and ATG.

| **Hydrogel** | **Conditions of processing** | **Self-healing time (mins)** |
| --- | --- | --- |
| ATG | In PBS  (hydrogen bonds intact) | 28.1 ± 2.9 |
| ATG + Urea | In 4M urea  (hydrogen bonds disrupted) | 47.4 ± 5.3 |

**Table S1.** The healing time of the hydrogel.

**
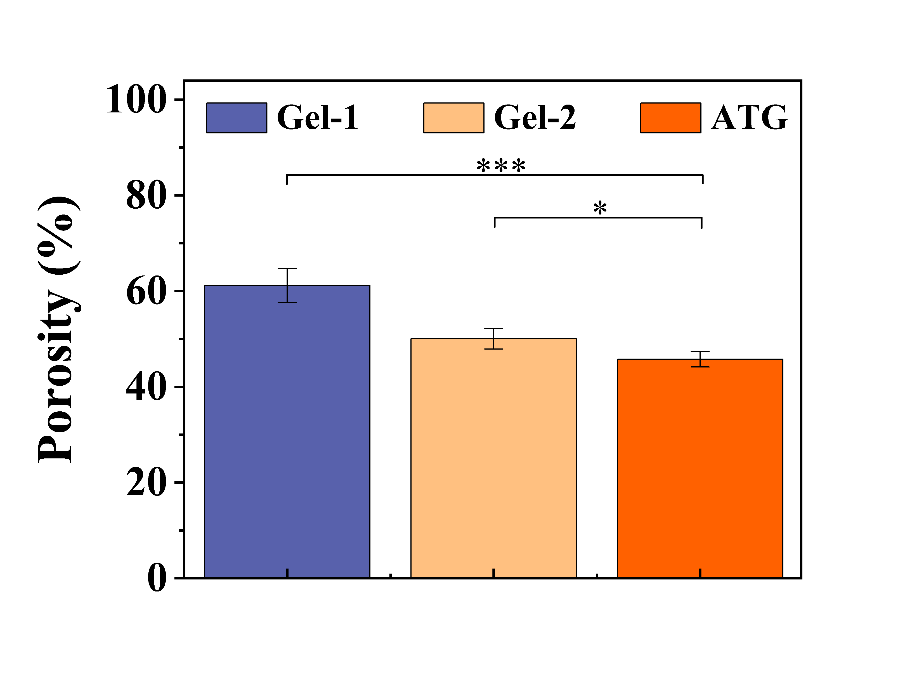
**

**Figure S6.** Porosity of hydrogels. (n = 3, Statistical differences: *p < 0.05, **p < 0.01, ***p < 0.001, and ns, not significant).


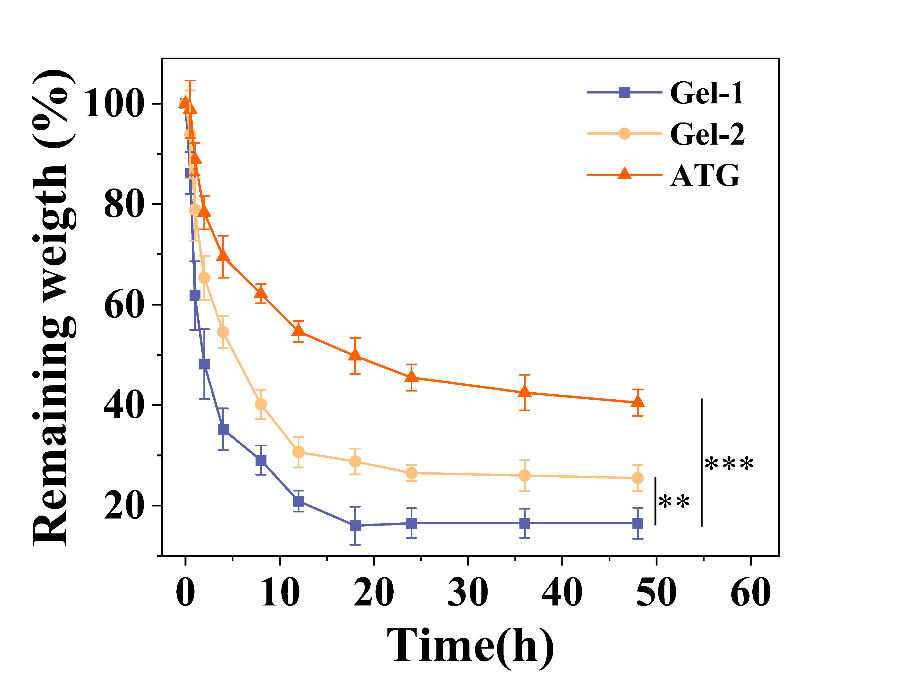


**Figure S7.** Degradation of hydrogels. (n = 3, Statistical differences: *p < 0.05, **p < 0.01, ***p < 0.001, and ns, not significant).


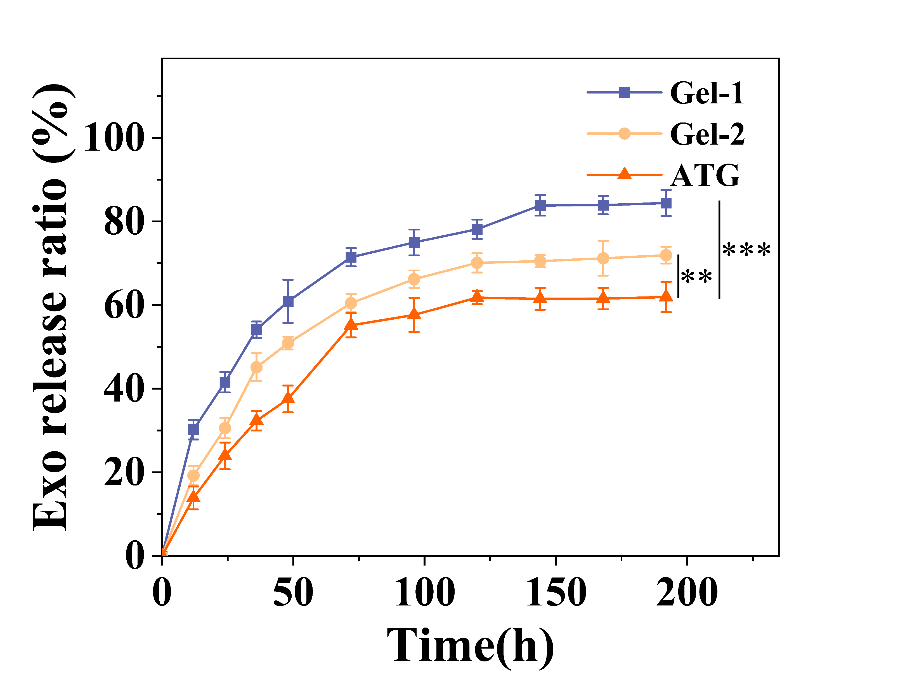


**Figure S8.** Exosome release of hydrogels. (n = 3, Statistical differences: *p < 0.05, **p < 0.01, ***p < 0.001, and ns, not significant).


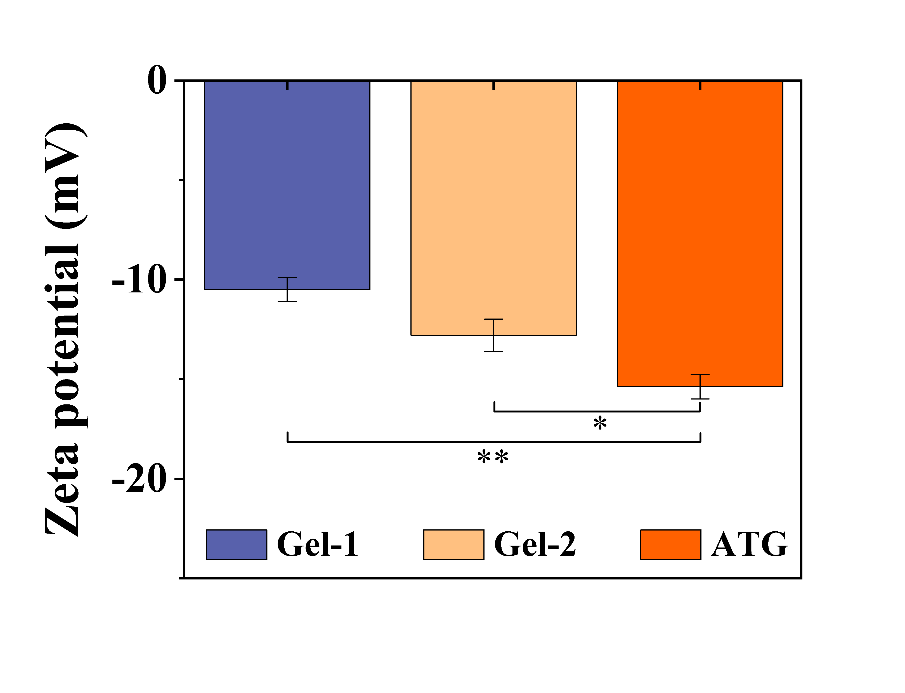


**Figure S9.** Zeta potential of hydrogels. (n = 3, Statistical differences: *p < 0.05, **p < 0.01, ***p < 0.001, and ns, not significant).


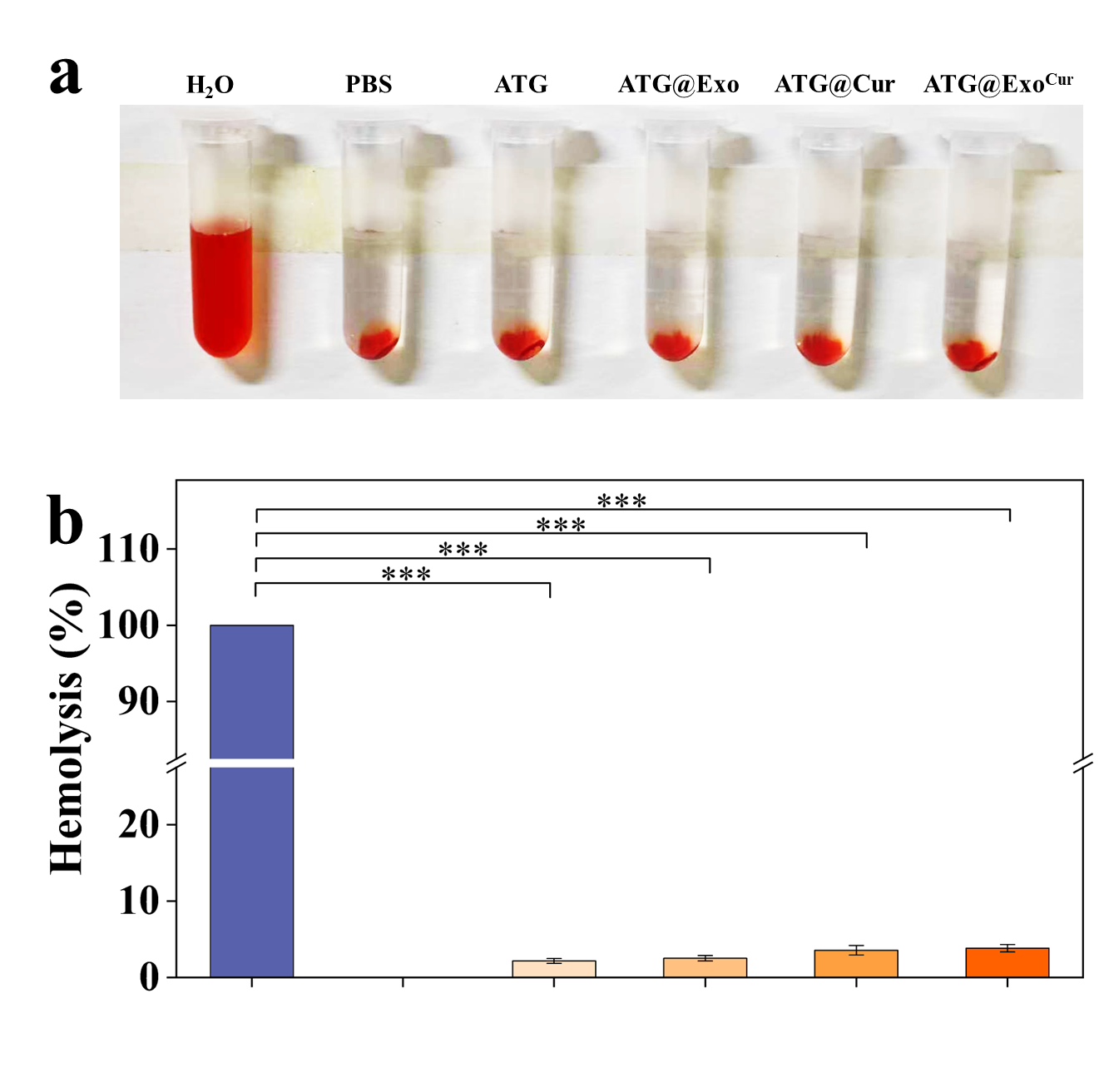


**Figure S10.** (a) Hemolysis in different experimental groups. (b) Quantitative statistics on hemolysis rates. (n = 3, Statistical differences: *p < 0.05, **p < 0.01, ***p < 0.001, and ns, not significant).


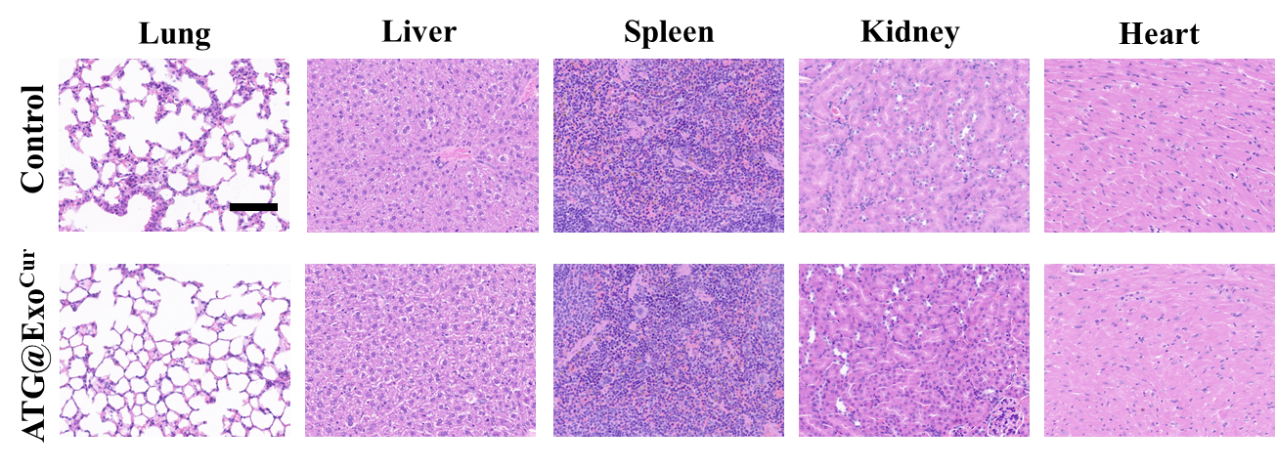


**Figure S11.** Organ compatibility of hydrogels. Scale bar is 100 μm.


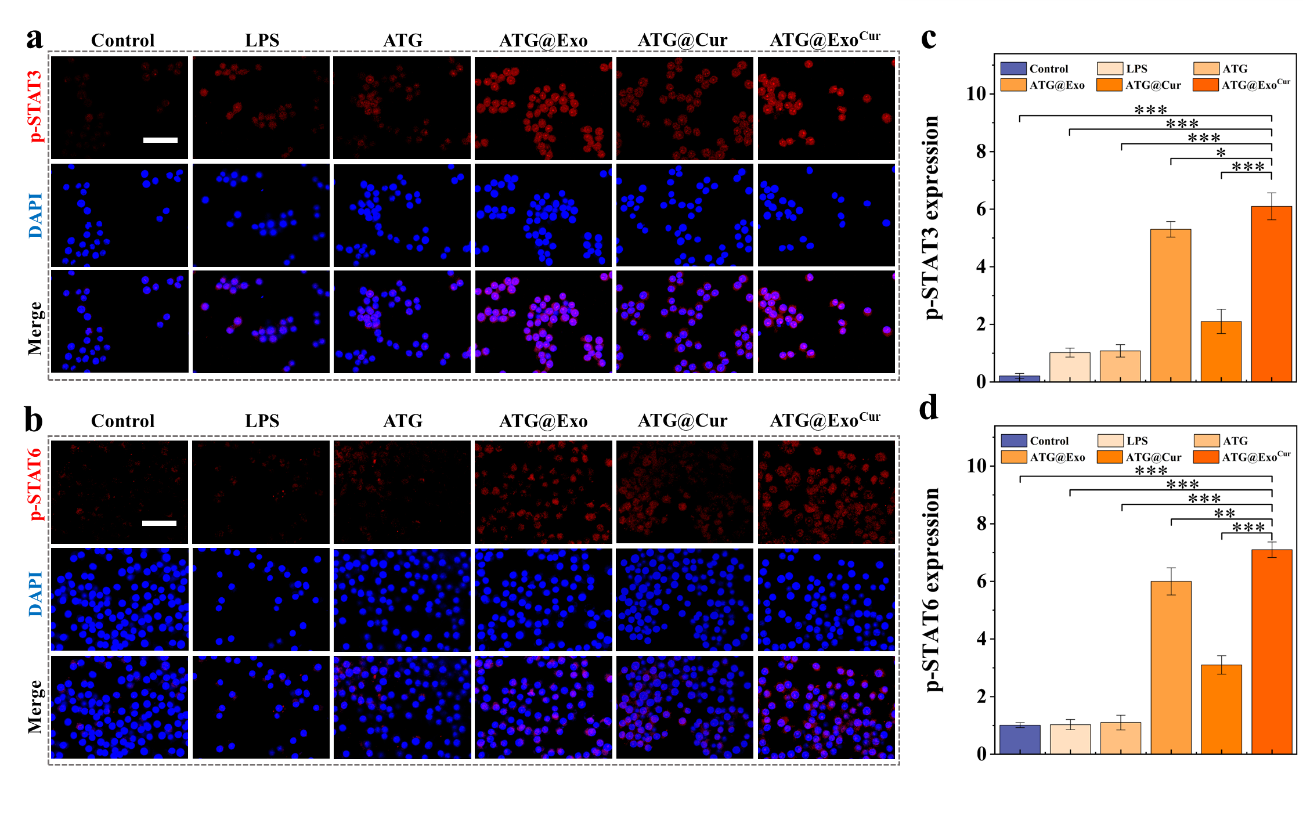


**Figure S12.** (a) Fluorescently stained p-STAT3 in macrophages. (b) Fluorescently stained p-STAT6 in macrophages. (c) Quantitative statistics on p-STAT3 expression levels. (d) Quantitative statistics on p-STAT6 expression levels. Scale bar is 50 μm. (n = 3, Statistical differences: *p < 0.05, **p < 0.01, ***p < 0.001, and ns, not significant).


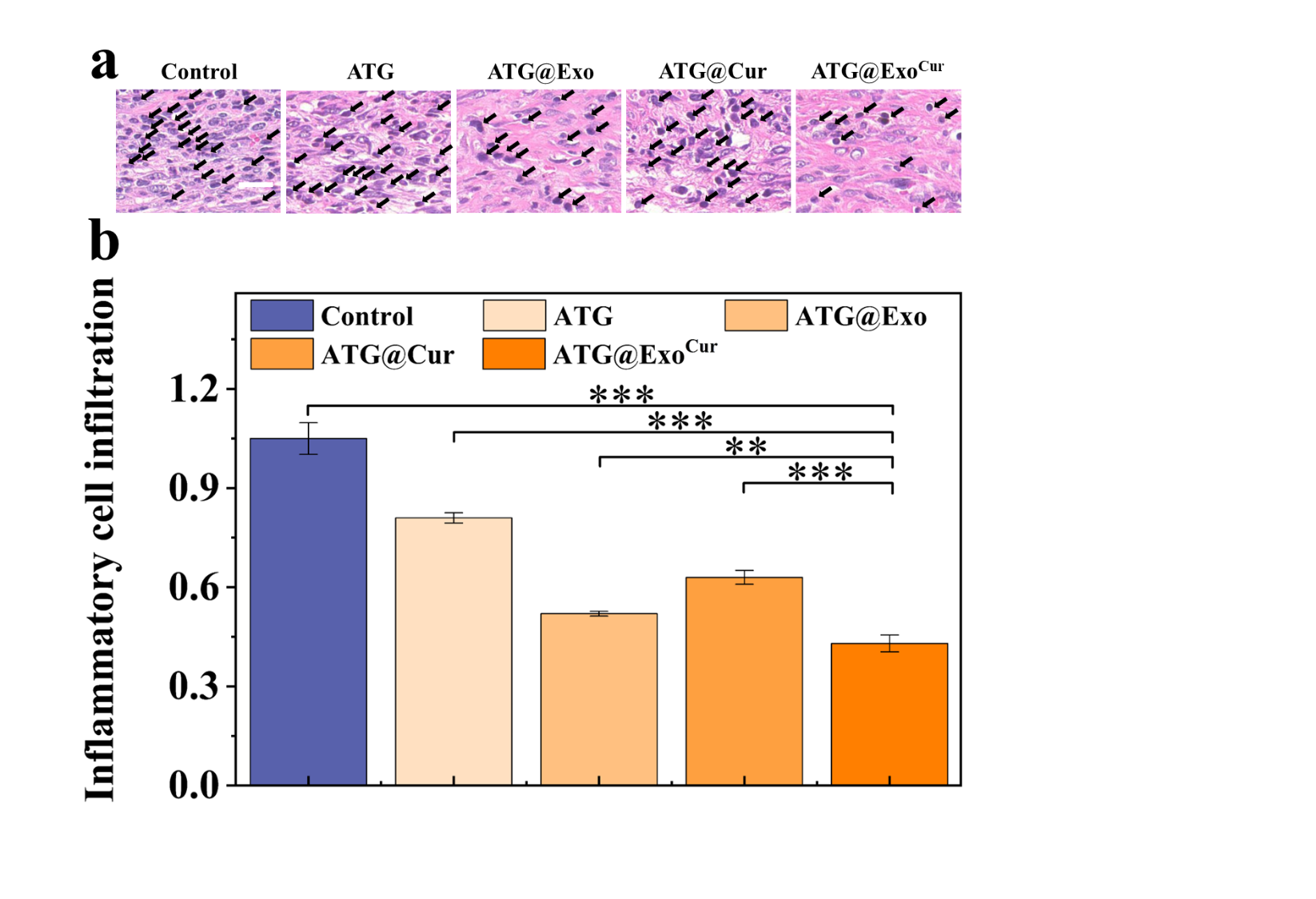


**Figure S13.** (a) Inflammatory cell infiltration in H&E-stained sections. (b) Quantitative statistics on inflammatory cell infiltration. Scale bar is 20 μm. (n = 5, Statistical differences: *p < 0.05, **p < 0.01, ***p < 0.001, and ns, not significant).
